# Synthesis and characterisation of biologically compatible TiO_2 _nanoparticles

**DOI:** 10.1186/1556-276X-6-423

**Published:** 2011-06-14

**Authors:** Richard W Cheyne, Tim AD Smith, Laurent Trembleau, Abbie C Mclaughlin

**Affiliations:** 1 The Chemistry Department, University of Aberdeen, AB24 3 UE, UK; 2 School of Medical Sciences, University of Aberdeen, AB25 2ZD, UK

## Abstract

We describe for the first time the synthesis of biocompatible TiO_2 _nanoparticles containing a functional NH_2 _group which are easily dispersible in water. The synthesis of water dispersible TiO_2 _nanoparticles coated with mercaptosuccinic acid is also reported. We show that it is possible to exchange the stearic acid from pre-synthesised fatty acid-coated anatase 5-nm nanoparticles with a range of organic ligands with no change in the size or morphology. With further organic functionalisation, these nanoparticles could be used for medical imaging or to carry cytotoxic radionuclides for radioimmunotherapy where ultrasmall nanoparticles will be essential for rapid renal clearance.

## Introduction

Organically functionalised inorganic nanoparticles are being increasingly studied as a result of their many technological applications. In particular, the synthesis of inorganic nanoparticles for biomedical applications is being widely researched. Biomedical applications of inorganic nanoparticles include biosensing [1], targeted drug delivery agents [2] and contrast agents in magnetic resonance imaging (MRI) [3,4]. Surface-coated superparamagnetic iron oxide nanoparticles have been extensively employed as magnetic resonance signal enhancers that can resolve the weakness of current MRI techniques. Most recently, it has been shown that by conjugating surface-coated Au-Fe_3_O_4 _nanoparticles to both herceptin and *cis*-platin, the nanoparticles can act as target-specific nanocarriers to deliver platin into Her2-positive breast cancer cells with strong therapeutic results [5]. Furthermore, these nanoparticles can act as both a magnetic and optical probe for tracking the platin complex in cells and biological systems. However, the iron oxide nanoparticles commonly used as MRI contrast agents have a radius of over 50 nm so that they have a limited extravasation ability and are subject to easy uptake by the reticuloendothelial system [6,7]. In order to enhance biological targeting efficiency, ultrasmall nanoparticles with greatly reduced hydrodynamic sizes are desired. Recently, ultrasmall (core size of 4.5 nm) c(RGDyK)-coated Fe_3_O_4 _nanoparticles have been synthesised [8], and results show a dramatic increase in cellular uptake. These nanoparticles were synthesised via thermal decomposition of Fe(CO)_5 _in the presence of the ligand 4-methycatechol (4-MC). The 4-MC-coated nanoparticles were then conjugated with a peptide c(RGDyK) via the Mannich reaction. There has been much research into the synthesis and properties of TiO_2 _nanoparticles since surface-modified TiO_2 _nanoparticles have many applications including photocatalysis [9] and photoelectric conversion [10,11]. Such research has shown that it is facile to make surface-coated TiO_2 _nanoparticles with an ultrasmall core size of 3 to 5 nm [12,13]. However, the study of TiO_2 _nanoparticles for biological applications, which have been shown to be non-toxic at low doses [14] (5 mg/kg body weight), has thus far been limited as such TiO_2 _nanoparticles are generally synthesised via a nonhydrolytic method and hence are non-dispersible in water. There are a couple of examples of functionalised TiO_2 _nanoparticles which are dispersible in water [15,16]; however, in these reports, a broad size distribution is evidenced (3 to 8 nm).

In this paper, we show that it is possible to synthesise ultrasmall TiO_2 _nanoparticles with a core size of 5 nm with a range of coated short-chain organic functional groups which are comparable in size to diabodies which exhibit rapid renal excretion [17]. The organically functionalised nanoparticles are highly dispersible in a range of solvents, and results show that when coated with aspartic acid or mercaptosuccinic acid, the nanoparticles are easily dispersible in water. Hence, for the first time, ultrasmall biocompatible TiO_2 _nanoparticles containing a functional NH_2 _or SH group have been synthesised. With further organic functionalisation and conjugation to a targeting moiety such as a single-chain antibody fragment or to biotin, these nanoparticles could be used to carry multiple short-lived radionuclides including ^99m^Tc and ^67^Ga for medical imaging or to cytotoxic radionuclides for radioimmunotherapy where ultrasmall nanoparticles will be essential for rapid renal clearance.

## Results and discussion

### Nanoparticle preparation

The two-phase thermal synthesis of titanium dioxide nanoparticles was adapted from a previously described procedure [13]. Typically, a solution of *tert-*butylamine dissolved in water was added to a Teflon-lined steel autoclave. Separately, titanium(IV) *n*-propoxide and stearic acid (SA) were dissolved in toluene and added to the autoclave. The autoclave was sealed and heated to 180°C for 16 h and allowed to cool to room temperature. TiO_2 _nanoparticles were recovered by precipitation with acetonitrile and isolated by filtration. The "SA-coated" nanoparticles are dispersible in chloroform and methanol but are not dispersible in water or acetonitrile. The approximate number of SA molecules bound to each nanoparticle core was calculated to be 500 by following an established procedure [12].

### Surface functionalisation

Exchange of the TiO_2_-bound stearic acid chains with various carboxylic acids was performed by reacting SA-coated nanoparticles with excess acids in refluxing chloroform. The resulting nanoparticles could be recovered by removal of solvent, re-suspension in acetonitrile, and filtration. The nanoparticles were dispersed in appropriate solvents, and nuclear magnetic resonance (NMR) spectra were taken. The degree of ligand exchange was determined by integration of the relevant signals of the distinct functional groups in the proton NMR spectra. The results are reported in Table 1. Approximately 37% of the stearic acid chains could be exchanged by benzoic acid (Benz) synthesised under these conditions. Exchange with phthalic acid led to the formation of non-dispersible nanoparticles, and the XRD powder pattern obtained indicates a large proportion of unbound phthalic acid that could not be removed. Synthesis of aspartic acid (Asp) and glycine (Gly) nanoparticles without the protective Boc group were unsuccessful, presumably due to the poor solubility of l-aspartic acid and glycine in chloroform. Only about 25% of the stearic acid chains could be exchanged by Boc-glycine (Boc-Gly). But ligand exchange with the bidentate ligands mercaptosuccinic acid (Mercapto) or Boc-aspartic acid (Boc-Asp) was almost quantitative as observed by proton NMR (^1^H NMR). The Boc group was later cleaved with 4 M HCl in dioxane. The resulting nanoparticles from both exchanges were easily dispersed in water (ca. 5 mg/ml), and the dispersion is stable for days without precipitation.

**Table 1:** Exchange of the TiO_2_-bound stearic acid chains with various carboxylic acids


**Entry**	**Carboxylic acid (ligand)**	**Ligand exchange (%)**
1		37^a,b^
2		20^c,d^
3		25^a,b ^(30)
4		>95^c,b ^(>95^e^)
5		>95^f^

^a^Determined by ^1^H NMR (400 MHz, CDCl_3_). ^b^XRD powder pattern indicated essentially pure nanoparticles. ^c^The nanoparticles were not dispersible in any solvent. ^d^Based on the recovery yield of ligand in acetonitrile. ^e^Determined by ^1^H NMR (400 MHz, D_2_O) after removal of the Boc group using HCl/dioxane (ammonium hydrochloride salt is obtained). ^f^Determined by ^1^H NMR (400 MHz, D_2_O)

### Characterisation of surface-functionalised nanoparticles

**Figure 1:**
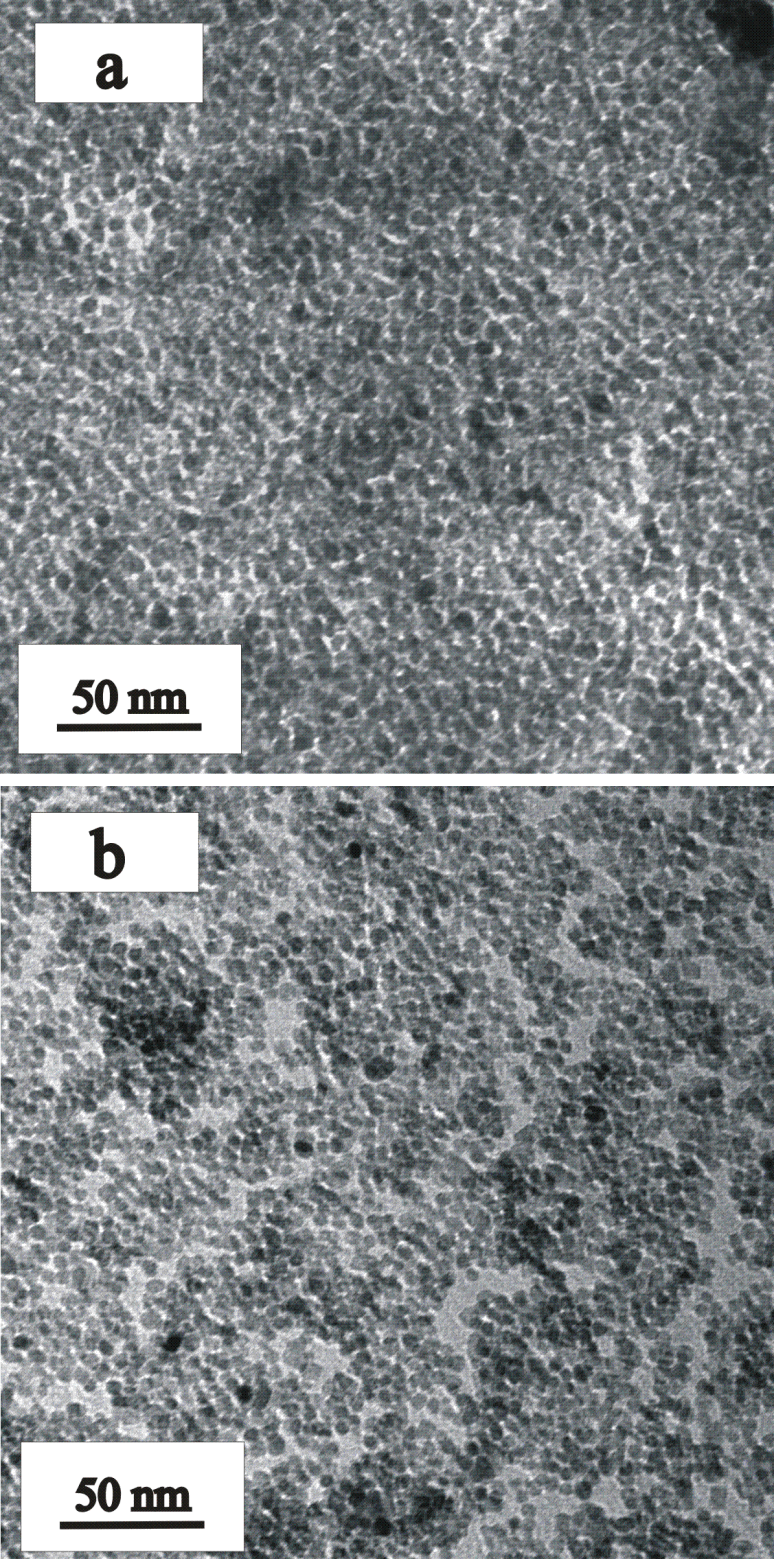
**TEM images of (a) SA-coated and (b) Asp-coated TiO_2 _nanoparticles**.

**Figure 2:**
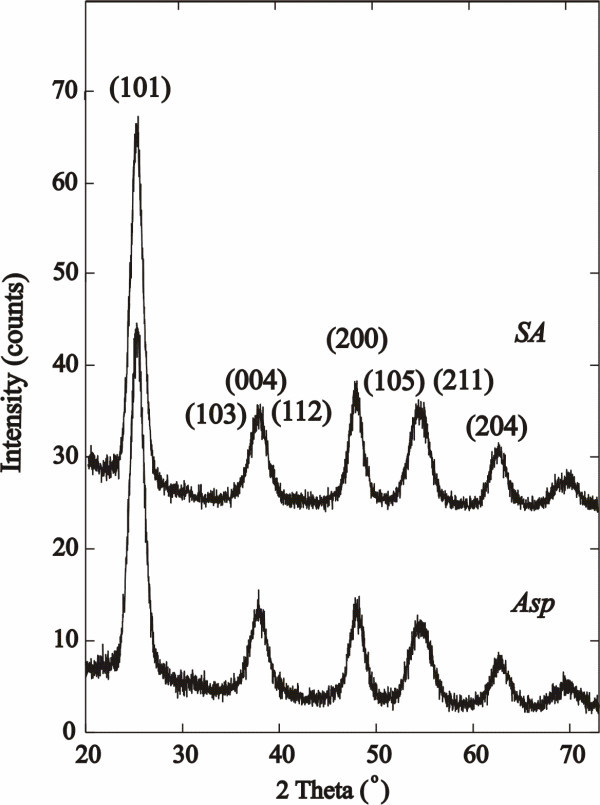
**XRD powder patterns of SA and Asp surface-coated TiO_2 _nanoparticles**. The patterns show formation of 5-nm anatase phase.

The TEM images of SA- and Asp-coated TiO_2 _nanoparticles are presented in Figure 1. The TEM images for the other coated nanoparticles and higher magnification images are displayed in the Additional file (Figures S1 and S2 in Additional file 1). The higher magnification shows that the nanoparticles prepared are spherical with a uniform diameter of 5 ± 1 nm, but that the nanoparticles agglomerate. Such agglomeration/aggregation of TiO_2 _nanoparticles is well documented and can be tuned by altering the pH (for example see references [9,18,19]). The mean hydrodynamic radius was determined using dynamic light scattering, and the results are displayed in Table 2 and confirm that when dispersed in solution, the coated TiO_2 _nanoparticles form agglomerates which vary in size from 141 to 601 nm. Powder X-ray diffraction (XRD) patterns of SA- and Asp-coated nanoparticles are shown in Figure 2. The diffraction patterns show that the anatase phase (JCPDS no. 21-1272) is formed, and the crystallite size was calculated at 5 nm using the Scherrer formula which is in good agreement with the TEM images [20]. The XRD patterns of the Benz, Boc-Gly, Boc-Asp, Mercapto and Gly surface-modified TiO_2 _nanoparticles are displayed in Figures S3 and S4 in Additional file 1. There is no change in particle size or crystal structure upon surface modification.

**Table 2:** Mean hydronamic radius for the different carboxylic acid-coated TiO_2 _nanoparticles determined from DLS measurements.

Carboxylic acid (ligand)	Mean hydrodynamic radius (nm)
SA	141
Mercapto	192
Asp	202
Gly	508
BA	601

**Figure 3:**
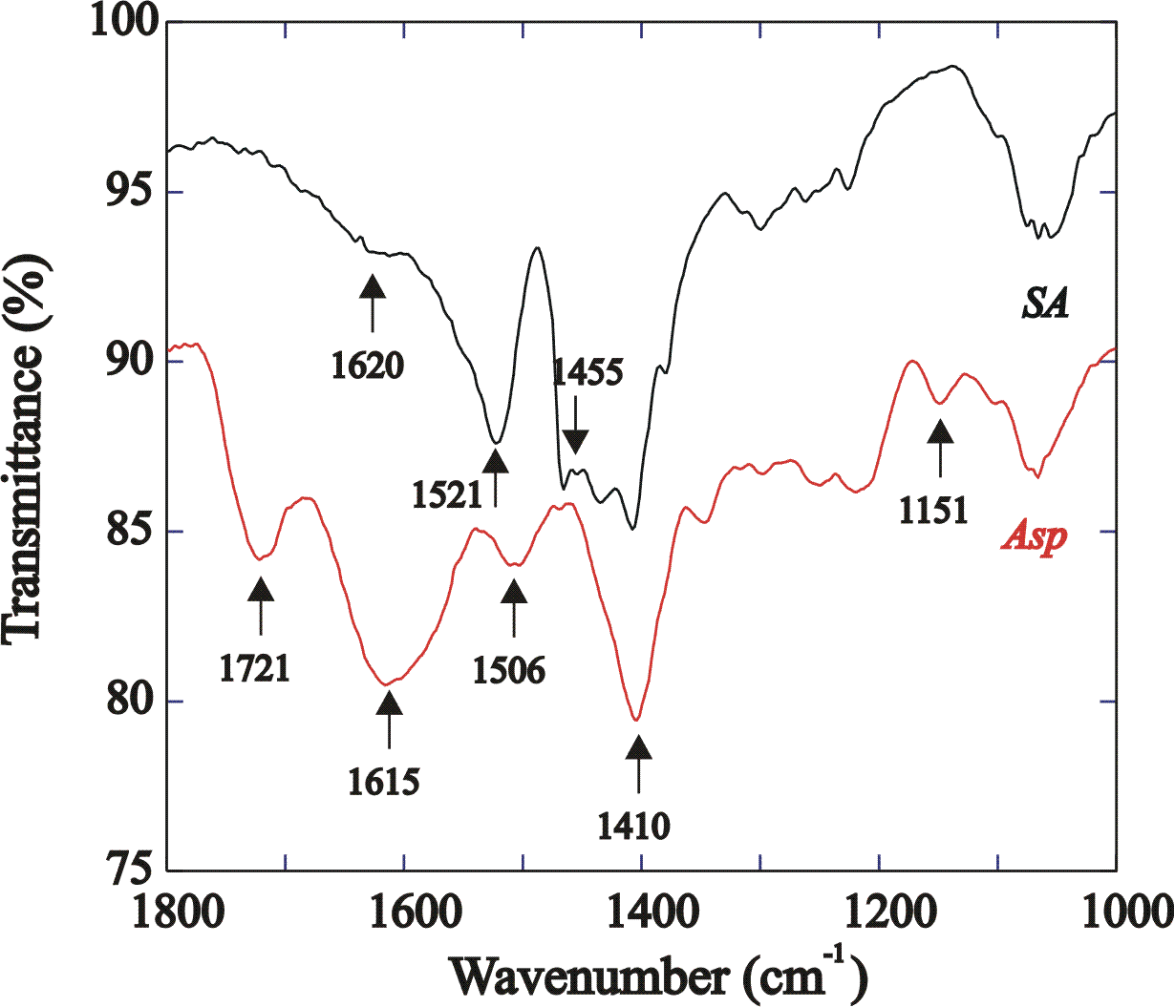
**Solid-state ATR-FTIR spectra of SA-coated (top) and Asp-coated (bottom) TiO_2 _nanoparticles**.

The presence of the various surface coatings were confirmed by Fourier transform infrared spectroscopy (FTIR) and ^1^H NMR measurements. The spectrum of pure stearic acid shows the C = O stretch vibration at 1,700 cm^-1^. This band is completely converted into three new bands in the spectrum of stearic acid-coated TiO_2 _nanoparticles as previously reported [12]. Two different carboxylate binding sites can be identified, a bridging complex (*ν*_a _*= *1,620 cm^-1^, *ν*_s _*= *1,455 cm^-1^) and a bidentate complex (*ν*_a _*= *1,521 cm^-1^, *ν*_s _*= *1455 cm^-1^). The infrared (IR) spectrum of the Benz-coated nanoparticles (Figure S5 in Additional file 1) shows no evidence of the free acid C = O stretch, and carboxylate peaks are detected at 1,630, 1,513 and 1,411 cm^-1^, while C = C aromatic stretches are detected at 1,599 and 1,448 cm^-1^. Upon ligand exchange with Boc-l-aspartic acid and subsequent removal of the Boc group, a change in the IR spectrum is evidenced (Figure 3). The carboxylate peaks shift to 1,506 and 1,410 cm^-1^, and the C-N stretching vibration is detected at 1,151 cm^-1^. The N-H bend is detected by the presence of the strong peak at 1,615 cm^-1^, demonstrating the presence of a primary amine; however, a C = O stretch observable at 1,721 cm^-1 ^suggests that not all of the carboxylate groups are bound to the TiO_2 _core. Two broad peaks are observed at 3,316 and 3,166 cm^-1 ^which correspond to N-H stretch peaks; the broadness of the peaks suggests H bonding interactions between adjacent molecules. The IR spectra of Benz-, Boc-Gly-, Boc-Asp-, Mercapto- and Gly-coated nanoparticles are displayed in Figure S5 in Additional file 1.

**Figure 4:**
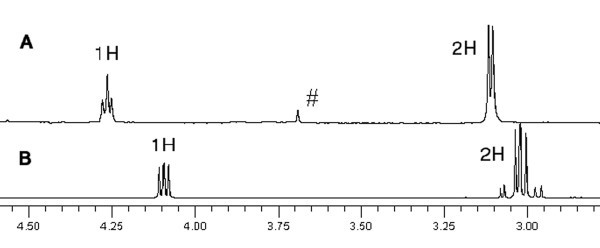
**Part of the ^1^H NMR spectrum (400 MHz) in D_2_O**. For Asp-coated nanoparticles (A) and free aspartic acid-coated nanoparticles (B). Number sign, residual dioxane from Boc deprotection.

**Figure 5:**
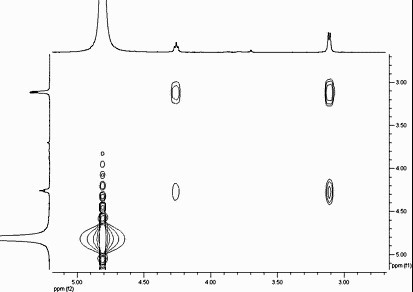
**2D COSY NMR spectrum (400 MHz, D_2_O) of aspartic acid-coated TiO_2 _nanoparticles**.

The Asp nanoparticles were further investigated by NMR. The proton NMR spectrum of free aspartic acid (Figure 4) shows a doublet of doublets at 4.09 ppm (^3^*J *= 4.4 Hz; ^3^*J *= 6.8 Hz) and two doublets of doublets at 3.05 ppm (^2^*J *= 18 Hz; ^3^*J *= 4.4 Hz) and 2.98 ppm (^2^*J *= 18 Hz; ^3^*J *= 6.8 Hz). For the aspartic acid-coated nanoparticles, these signals are significantly shifted downfield (0.05 to 0.17 ppm) and they are slightly broadened. Curiously, the geminal coupling constant for the CH_2 _group has apparently disappeared as the CH group appears as a triplet (*J *= 5.6 Hz) and the CH_2 _group appears as a doublet (*J *= 5.2 Hz). Since the two methylene hydrogens are diastereotopic, the most likely explanation to this anomaly is that the chemical environment of both nuclei is such that they have almost identical chemical shifts. The discrepancy in the coupling constants (5.6 versus 5.2 Hz) can be explained by the signals given by the doublet and triplet appearing slightly broad. A two-dimensional (2D) correlation spectroscopy (COSY) experiment on these nanoparticles confirmed this coupling (Figure 5). The strong correlation clearly seen between the CH triplet (4.25 ppm) and the CH_2 _doublet (3.09 ppm) indicates that despite the unusual coupling constants obtained from the ^1^H NMR, the nuclei in question are spin coupled. This validates their identities and indicates that the nanoparticle contains aspartic acid as a ligand albeit in a slightly altered chemical state to that of the free acid.

## Conclusions

In summary, we have created a facile route to synthesise ultrasmall surface-coated TiO_2 _nanoparticles with a range of organic coatings. Furthermore, the surface-coated nanoparticles are incredibly robust so that it is possible to perform ligand exchange reactions on the outer capping groups without disturbing the overall size or structure morphology of the nanoparticles. Results suggest that ligand exchange is most successful with bidentate ligands as a result of the availability of two carboxylic acid groups which bind to the TiO_2 _core.

This two-step approach toward the synthesis of surface-modified TiO_2 _nanoparticles allows for fine tuning of the nanoparticle core size in the first step before surface modification with suitable ligands in the second. By separating the surface modification step from that of the nanoparticle formation, this method allows for the production of identical nanoparticle cores before differentiation by surface modifications. Additionally, the use of bifunctional ligands to form the nanoparticle coating allows for the possibility of post-synthesis modifications to further functionalise the nanoparticle. This may be beneficial for use in biological applications as the initial surface functionalisation can convey improved water solubility before addition of more biologically relevant moieties. With further organic functionalisation and conjugation to a targeting moiety, the biological applications of the nanoparticles described here include the transport of multiple short-lived radionuclides including ^99^Tc and ^67^Ga for medical imaging or to cytotoxic radionuclides for radioimmunotherapy. The biological potential of these new nanostructures is currently being investigated.

## Experimental procedures

### General

All ligand exchange reactions were performed under an argon atmosphere. All reagents were purchased from Sigma-Aldrich (Sigma-Aldrich Company Ltd, Dorset, England) and used without further purification. Cleavage of Boc protecting groups was achieved by stirring in 4 M HCl/dioxane for 3 h under argon.

### Analytical measurements

Routine ^1^H NMR and COSY data for TiO_2 _nanoparticles were obtained at 400 MHz on a VarianUnity INOVA instrument (Agilent Technologies Ltd, UKInfrared spectra were obtained from 400 scans at 4 cm^-1 ^resolution using a Nicolet 380 spectrometer (Thermo Electron Corporation, Franklin, MA, USA) fitted with a diamond attenuated total reflectance (ATR) platform. IR and NMR data reported were obtained at room temperature. Room temperature X-ray diffraction patterns were collected for the organically coated TiO_2 _nanoparticles on a Bruker D8 Advance diffractometer (Bruker AXS Ltd, Coventry, UK) with twin Gobel mirrors using Cu Kα_1 _radiation. Data were collected over the range 20° < 2*θ *< 80°, with a step size of 0.02°. Transmission electron microscopy images were obtained for the organically coated TiO_2 _nanoparticles on a Philips CM10TEM (FEI Ltd, Netherlands). Dynamic light scattering (DLS) was performed using a Malvern mastersizer (Malvern Instruments Ltd, Malvern, UK).

### Synthesis of titanium dioxide nanoparticles

Titanium dioxide nanoparticles were synthesised by a two-phase thermal approach adapted from a previously described procedure [13]. Typically, a solution of 0.15 mL of *tert-*butylamine (1.43 mmol) dissolved in 14.5 mL of water was added to a 45-mL Teflon-lined steel autoclave. Separately, 0.225 g of titanium(IV) *n*-propoxide (0.792 mmol) and 0.75 g of stearic acid (2.64 mmol) were dissolved in 14.5 mL of toluene and added to the autoclave without additional stirring. The autoclave was sealed and heated to 180°C for 16 h and allowed to cool to room temperature. The TiO_2 _nanoparticles were recovered by precipitation with 90 mL of acetonitrile and isolated by filtration. Off-white solid; ^1^H NMR (CDCl_3_); *δ *0.88 (*t*, 3H), 1.25 (*s*, 30H) and 2.03 (*s*, 2H); IR *ν*_max _2,960, 2,915, 2,848, 1,620, 1,521, 1,455, 1,400, 1,300, 1,258, 1,220 and 1, 066 cm^-1^.

### Procedure for surface modification of nanoparticles

A solution of carboxylic acid (150 mg) in 5 mL chloroform was added to a reaction vessel containing a dispersion of "SA-coated" TiO_2 _nanoparticles (100 mg) in 10 mL chloroform. The reaction was stirred for 18 h under reflux. The resultant surface-modified nanoparticles were recovered by evaporation of the solvent *in vacuo*, re-suspension in acetonitrile and filtration. Unbound starting material was removed by repeated washings of the nanoparticles with acetonitrile.

### Benzoic acid exchanged TiO_2_

Off-white solid; 86% yield; ^1^H NMR indicates an incomplete exchange (37%) of stearic acid with benzoic acid; ^1^H NMR (CDCl_3_); *δ *0.88 (*t*, 3H), 1.28 (*s*, 28H), 1.65 (*t*, 2H), 2.34 (*t*, 2H), 7.42 (*t*, 1.2H), 7.53 (*t*, 0.6H) and 8.06 (*d*, 1.2H); IR *ν*_max _2,956, 2,919, 2,849, 1,630, 1,599, 1,513, 1,448 and 1,411 cm^-1^.

### Glycine exchanged TiO_2_

Synthesis was performed from Boc-glycine. Cleavage of the protecting group was achieved by stirring the resulting nanoparticles under argon in 4 M HCl/dioxane for 3 h. Off-white solid; 91% yield; ^1^H NMR indicates an incomplete exchange (30%) of stearic acid with glycine; ^1^H NMR (CDCl_3_); *δ *0.88 (*t*, 3H), 1.25 (*s*, 30H), 2.02 (*d*, 2H), 2.33 (*s*, 1H), 3.75 (*s*, 1.4H); IR *ν*_max _3,319, 3,115, 2,991, 2,928, 1,742, 1,613, 1,495, 1,435, 1,406, 1,337, 1,305, 1,248, 1,118, 1,066 and 901 cm^-1^.

### Aspartic acid exchanged TiO_2_

Synthesis was performed from Boc-aspartic acid. Cleavage of the protecting group was achieved by stirring the resulting nanoparticles under argon in 4 M HCl/dioxane for 3 h. Off-white solid; >95% yield; ^1^H NMR (D_2_O); *δ *1.40 (*s*, 0.4H), 2.03 (*s*, 0.4H), 2.13 (*s*, 0.3H), 3.09 (*d*, 2H, *J *= 5.2 Hz), 4.25 (*t*, 1H, *J *= 5.6 Hz); COSY clearly shows coupling between the protons of the doublet (*δ *3.09) and triplet (*δ *4.25); IR *ν*_max _3,316, 3,166, 2,970, 2,910, 1,721, 1,615, 1,506, 1,410, 1,346, 1,296, 1,253, 1,220, 1,151 and 1,066 cm^-1^.

### Phthalic acid exchanged TiO_2_

Off-white solid; purification not possible; resulting nanoparticles not dispersible.

### Mercaptosuccinic acid exchanged TiO_2_

Synthesis was performed using mercaptosuccinic acid. To reduce the possibility of oxidation occurring between mercaptosuccinic acid moieties, the reaction was performed under anhydrous conditions but in an otherwise identical manner to previous exchange reactions. Pale-yellow solid; >95% yield; ^1^H NMR (D_2_O); *δ *2.62 (*m*, 1H) and 2.91 (*m*, 1H); IR *ν*_max _2,915, 2,848, 1,685, 1,535, 1,515, 1,442 and 1,384 cm^-1^.

## Competing interests

The authors declare that they have no competing interests.

## Authors' contributions

ACM, LT and TADS designed the study; RC performed the experiments with help from ACM, LT and TADS; All authors contributed to drafting the manuscript; All authors edited and approved the manuscript.

## Supplementary Material

Additional file 1 **Supplementary data**. X-ray diffraction, TEM and spectroscopic data for coated titanium nanoparticles. Click here for file
