# Supplementary material for: Synthesis and characterisation of biologically compatible TiO2 nanoparticles
Source: Nanoscale Res Lett. 2011 Jun 14;6(1):423. doi: 10.1186/1556-276X-6-423 (PMC3211840; doi:10.1186/1556-276X-6-423)
Supplement: Additional file 1 — Supplementary data. X-ray diffraction, TEM and spectroscopic data for coated titanium nanoparticles. [file 1556-276X-6-423-S1.DOC]

Supporting information for:

**Synthesis and Characterisation of Ultrasmall Biologically compatible TiO_2_ Nanoparticles**

**Richard W. Cheyne^1,2^, Tim A. D. Smith^2^, Laurent Trembleau^1^ and Abbie C. Mclaughlin^1*^**

^1^ The Chemistry Department, University of Aberdeen, Meston Walk, Aberdeen, AB24 3 UE

^2^ Institute of Medical Sciences, University of Aberdeen, Foresterhill, Aberdeen, AB25 2ZD

**X-ray diffraction, TEM and spectroscopic data for coated titanium nanoparticles**

**
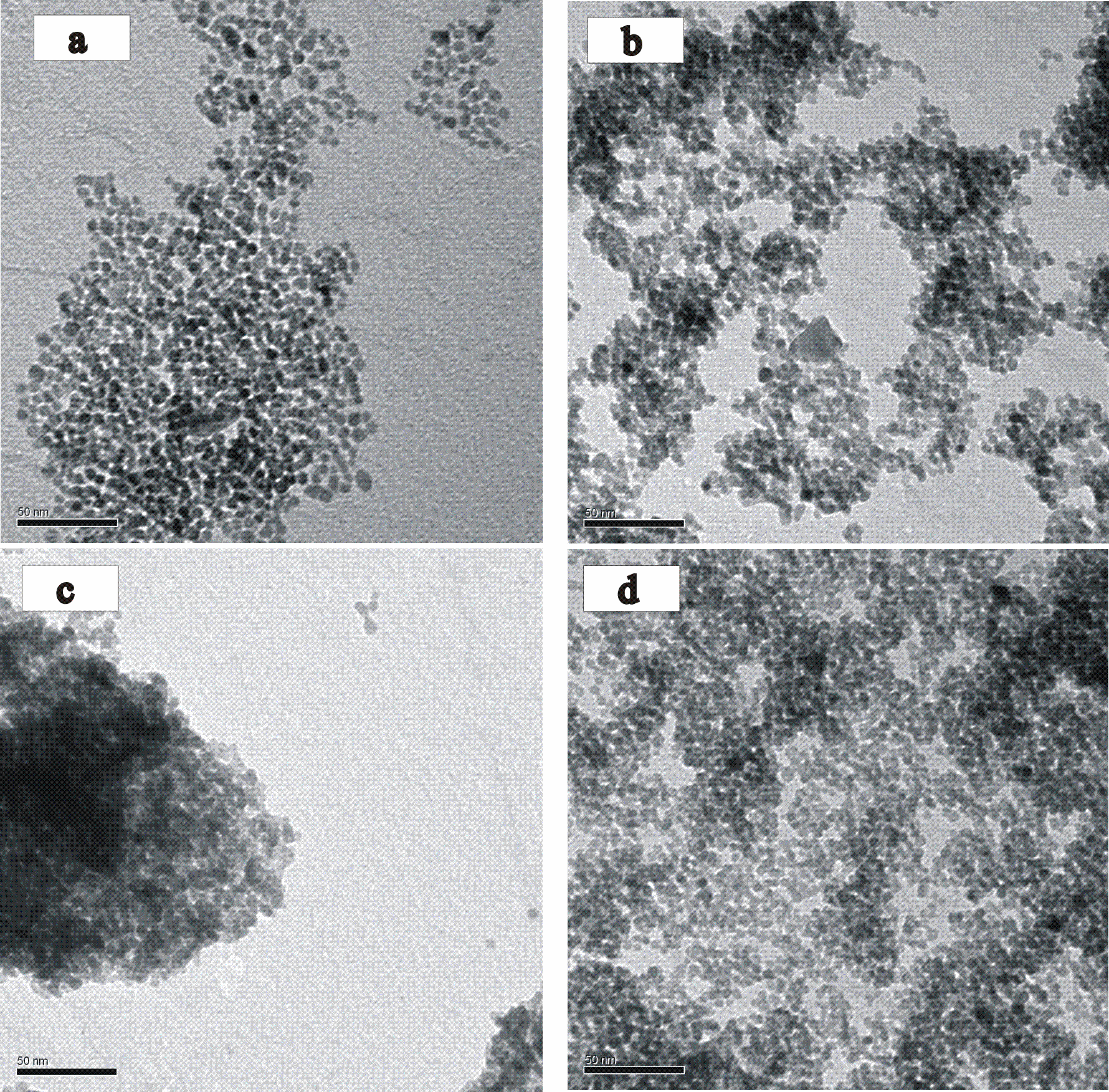
**

**S1** TEM images of a) *Benz*, b) *Boc-Gly*, c) *Boc-Asp* and d) *Gly* coated TiO_2_ nanoparticles.


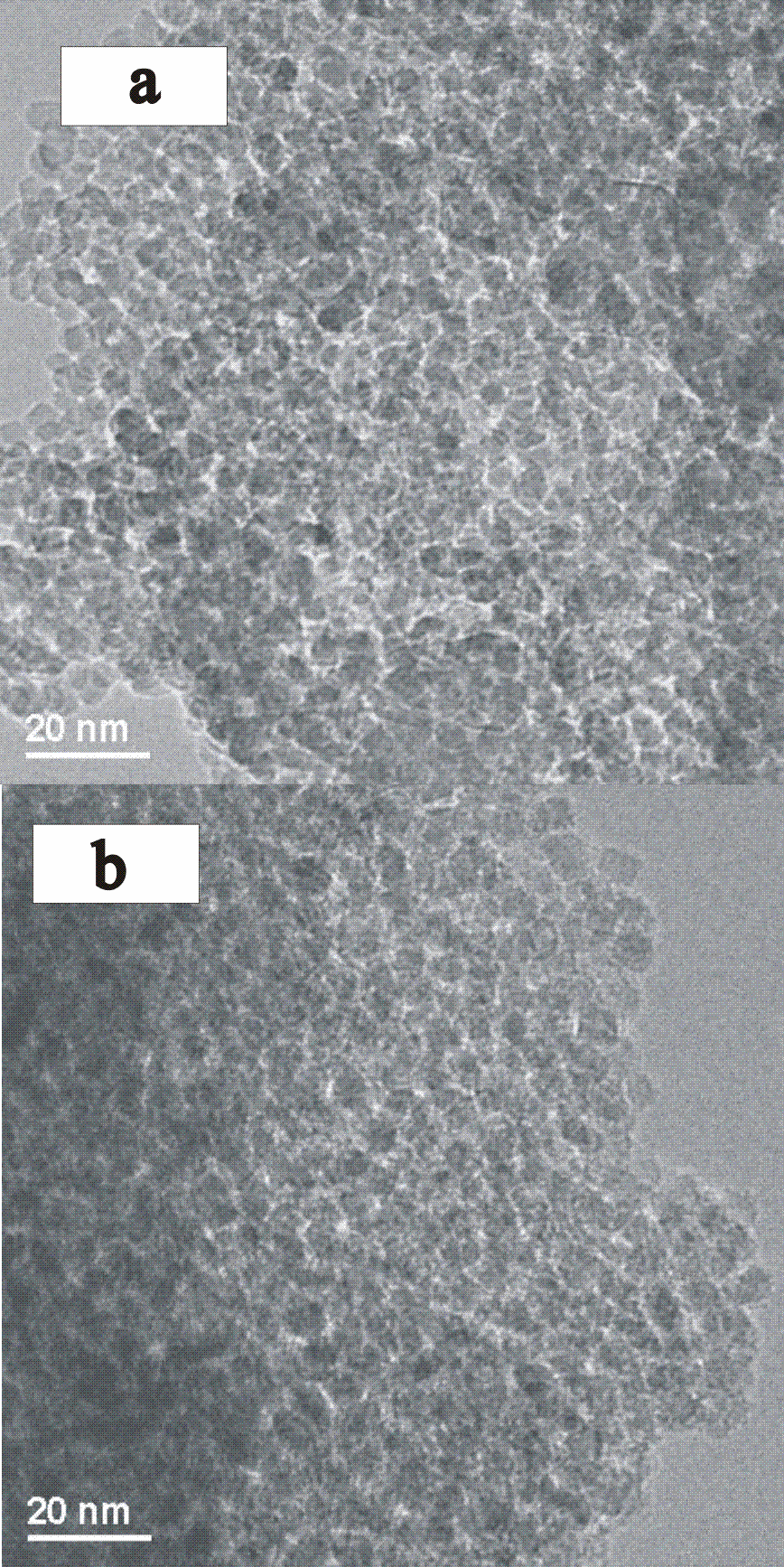


**S2** Higher magnification TEM images of a) *Mercapto*, and b) *Asp* coated TiO_2_ nanoparticles.


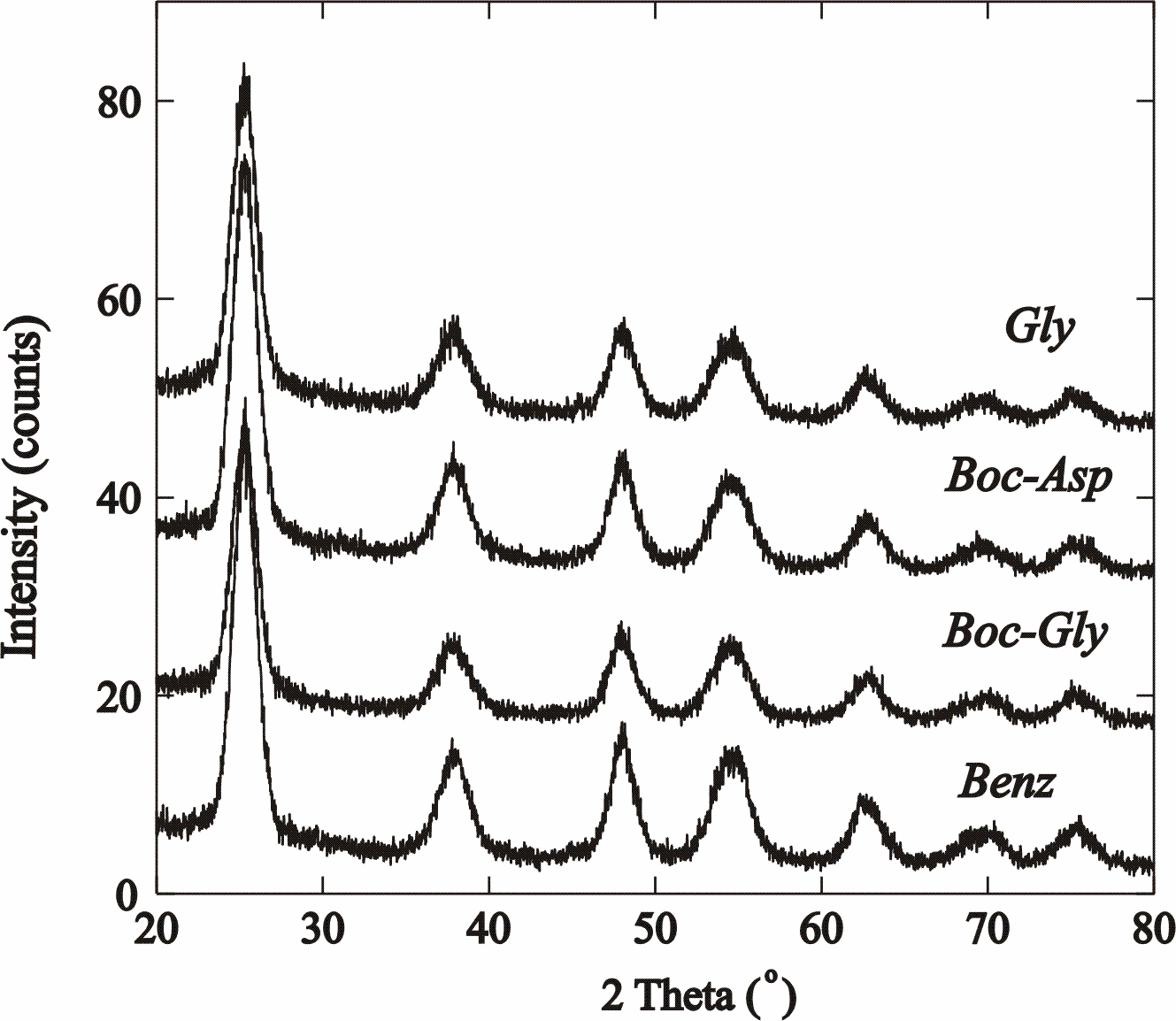


**S3** XRD powder patterns of surface coated TiO_2_ nanoparticles showing formation of 5nm anatase phase.


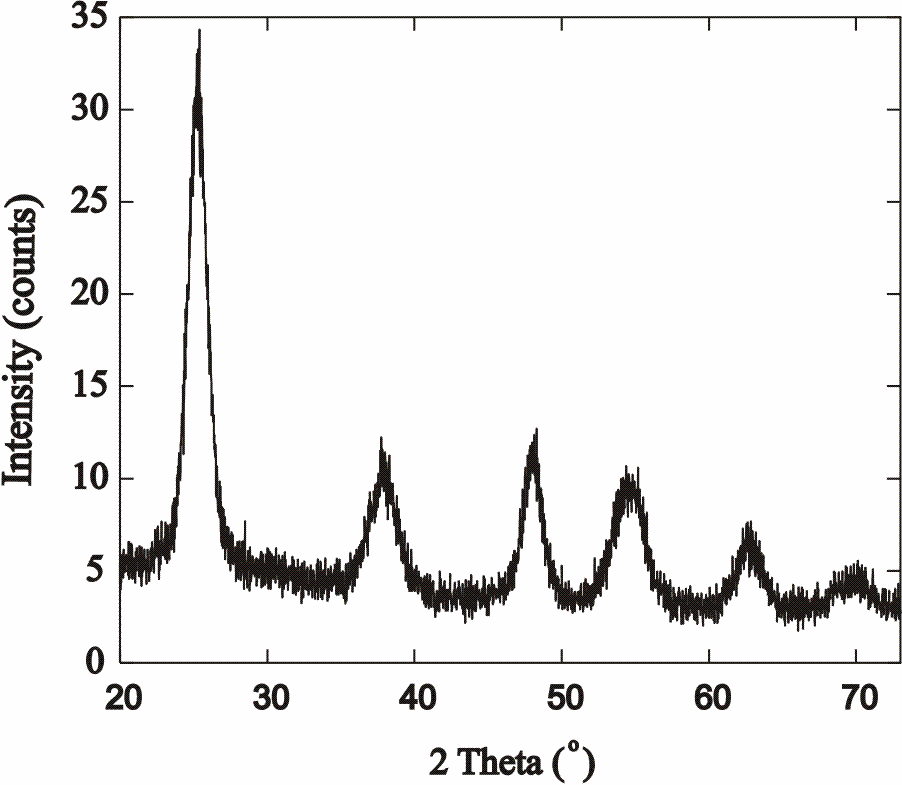


**S4** XRD powder patterns of *Mercapto* surface coated TiO_2_ nanoparticles showing formation of 5nm anatase phase.

**
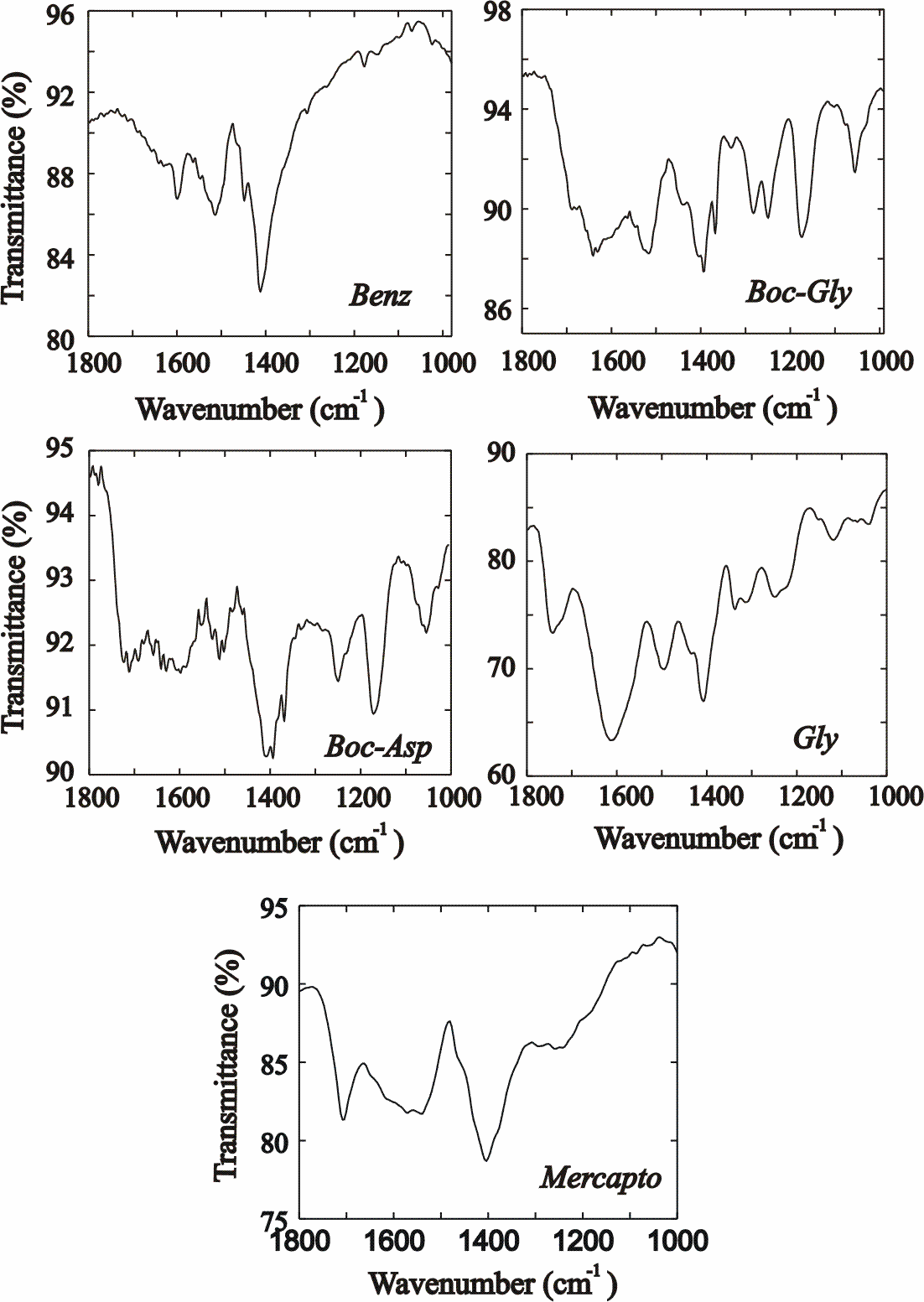
**

**S5** Solid state ATR-FTIR spectra of the coated TiO_2_ nanoparticles.
